# Linear and Non-linear Dynamic Methods Toward Investigating Proprioception Impairment in Non-specific Low Back Pain Patients

**DOI:** 10.3389/fbioe.2020.584952

**Published:** 2020-11-30

**Authors:** Seyed Mohammadreza Shokouhyan, Mehrdad Davoudi, Maryam Hoviattalab, Mohsen Abedi, Soha Bervis, Mohamad Parnianpour, Simon Brumagne, Kinda Khalaf

**Affiliations:** ^1^Department of Mechanical Engineering, Sharif University of Technology, Tehran, Iran; ^2^Physiotherapy Research Center, School of Rehabilitation, Shahid Beheshti University of Medical Sciences, Tehran, Iran; ^3^Physical Therapy Department, School of Rehabilitation Sciences, Shiraz University of Medical Sciences, Shiraz, Iran; ^4^Rehabilitation Sciences Research Center, Shiraz University of Medical Sciences, Shiraz, Iran; ^5^Department of Rehabilitation Sciences, KU Leuven, Leuven, Belgium; ^6^Healthcare Engineering Innovation Center, Department of Biomedical Engineering, Khalifa University of Science and Technology, Abu Dhabi, United Arab Emirates

**Keywords:** posture control, low back pain, COP, proprioception, recurrence quantification analyses, vibrator

## Abstract

Central nervous system (CNS) uses vision, vestibular, and somatosensory information to maintain body stability. Research has shown that there is more lumbar proprioception error among low back pain (LBP) individuals as compared to healthy people. In this study, two groups of 20 healthy people and 20 non-specific low back pain (NSLBP) participants took part in this investigation. This investigation focused on somatosensory sensors and in order to alter proprioception, a vibrator (frequency of 70 Hz, amplitude of 0.5 mm) was placed on the soleus muscle area of each leg and two vibrators were placed bilaterally across the lower back muscles. Individuals, whose vision was occluded, were placed on two surfaces (foam and rigid) on force plate, and trunk angles were recorded simultaneously. Tests were performed in eight separate trials; the independent variables were vibration (four levels) and surface (two levels) for within subjects and two groups (healthy and LBP) for between subjects (4 × 2 × 2). MANOVA and multi-factor ANOVA tests were done. Linear parameters for center of pressure (COP) [deviation of amplitude, deviation of velocity, phase plane portrait (PPP), and overall mean velocity] and non-linear parameters for COP and trunk angle [recurrence quantification analysis (RQA) and Lyapunov exponents] were chosen as dependent variables. Results indicated that NSLBP individuals relied more on ankle proprioception for postural stability. Similarly, RQA parameters for the COP on both sides and for the trunk sagittal angle indicated more repeated patterns of movement among the LBP cohort. Analysis of short and long Lyapunov exponents showed that people with LBP caused no use of all joints in their bodies (non-flexible), are less stable than healthy subjects.

## Introduction

Non-specific low back pain (NSLBP) is a highly prevalent public health challenge with severe health and economic consequences worldwide. 60 to 80 percent of the world’s population experience at least one episode of low back pain (LBP) in their life time (Waddell, 1987; Burton et al., 1995; Méndez and Gómez-Conesa, 2001; Truchon, 2001), with 15% reporting pain in the acute range (Liebenson, 1996). Overall, the documented monthly prevalence of LBP is estimated around 23.2% (Hoy et al., 2012). Although LBP is very common among people between the ages of 35–55 (Sarker et al., 2017), it impacts individuals of all ages. Indeed, reports indicate that low back pain represents a prevalent limiting physical factor for adults under 45 years of age, and is considered as the most common cause of job-related disability and a key contributor to missed work days (Hart et al., 1995; Praemer et al., 1999). The cost of treating patients with low back pain has major economic implications (Hashemi et al., 1997, 1998; Filiz et al., 2005). In the United States alone, the total cost associated with LBP healthcare ranges from $84 billion to $624 billion annually including indirect costs due to the loss of revenue and reduced productivity (Katz, 2006; Rubin, 2007; Dagenais et al., 2008). Importantly, prevalence of LBP has increased by more than 50% since 1990, and is projected to continue to increase specially in low and middle income countries (LMICs) where resources are limited and the lifestyle is rapidly becoming more sedentary (Clark and Horton, 2018). Recently, several studies have been conducted to determine the causes of low back pain (Allegri et al., 2016), however, further prospective studies are needed to identify the potential risk factors for developing low back pain.

Although postural control for LBP patients is an active area of research, many questions remain unanswered, particularly in terms of changes in sensory input and proprioception. In terms of the physiological processes associated with postural control, it is assumed that once the human neuronal control system senses a deviation associated with the trunk reference location, it sends commands for producing corrective ankle torque to counteract such deviations. This process, however, is highly dependent on the integrity of the three sensory systems: the vision, vestibular, and somatosensory systems. It is likely that the disruption of any one of these systems would negatively impact the final output of the postural system. Injuries or medical conditions that affect the neuromuscular system, such as stroke, muscle weakness, and other psychological factors including anxiety can be responsible for disturbing the function of the sensory systems (Jamali et al., 2019; Bervis et al., 2020). Another factor which could disturb the integrity of the sensory system is NSLBP, which has been shown to affect postural control. Prior investigations have observed decreased postural control/robustness during standing, especially when the standing task becomes more complicated, such as standing on unstable surfaces (Mientjes and Frank, 1999; Mok et al., 2004; della Volpe et al., 2006; Bervis et al., 2020). Impaired proprioception has been suggested as a possible mechanism which causes impairment in postural control, although we do not seek a particular assessment of proprioception impairment in this study.

The proprioception sensory system or central processing of proprioception information may be impaired in individuals with chronic low back pain (della Volpe et al., 2006). It should be noted, however, that the compromised delivery of proprioceptive information does not necessarily disturb the postural function of a person with LBP as he/she may still have sufficient motor control to overcome the deficit. Nonetheless, a disturbed sense of proprioception in people with LBP could impact their ability to control postural response (della Volpe et al., 2006), particularly when the complexity of postural conditions increases [e.g., walking on unstable or uneven surfaces, standing on one leg, rapid movements of the upper limb (bending), whole body vibration (X), etc.], As such, postural fluctuations and consequent postural control adaptation strategies are likely to significantly increase in LBP patients (della Volpe et al., 2006).

Brumagne et al. (2008) indicated that individuals without LBP are more reliant on ankle proprioception while standing on an unstable surface as compared to standing on a stable surface. In contrast, non-specific low back pain (NSLBP) patients exhibit similar levels of reliance on ankle proprioception regardless of stability conditions. Thus, the ability to discriminately employ ankle proprioception strategy is decreased in NSLBP individuals. Similarly, Claeys et al. (2011) reported decreased variability in postural control strategies among LBP patients during standing and sitting conditions. They found that young people without LBP are able to choose an optimal strategy for postural control based on postural conditions, while conversely, young adults with NSLBP shows reduced variability in self-selected proprioception control strategies. Claeys et al. (2012) also evaluated the variability in proprioception during sitting and rising movements, demonstrating that people with low back pain used less lumbar proprioception to control posture in comparison to their healthy counterparts. Claeys et al. (2015) further examined the potential impact of strategy change for LBP risk, with findings indicating that a higher reliance on ankle-steered proprioception elevated the risk for mild NSLBP. In contrast, fluctuations in postural angle, psychological variables, and physical activity levels did not increase the risk for LBP among the study’s cohort. In a recent study, we investigated the classification of NSLBP patients using specific questionnaires (Davoudi et al., 2020). We have also explored the effect of rehabilitation tools (such as the flexi bar) on muscle activation in NSLBP patients (Bervis et al., 2020). The current work expands our previous research by describing a methodology to quantitatively characterize postural control in NSLBP patients based on various advanced linear and non-linear dynamic analysis tools (Linear variability, RQA and Lyapunov exponents). In particular, this study aims to quantify and compare proprioception control parameters (body sway and stability) between non-specific low back pain patients and healthy controls. Our hypothesis is that LBP patients are challenged in the optimal use of their proprioception signals which leads to diminished postural control during normal activities.

## Materials and Methods

### Ethics

The studies involving human participants were reviewed and approved by the University Internal Ethics Board (approved by IRB of Shahid Beheshti University of Medical Sciences, Tehran, IR, No: IR.SBMU.RETECH.REC.1396.1392). The participants provided their written informed consent to participate in this study. Written informed consent was obtained from the individuals for the publication of any potentially identifiable images or data included in this article.

### Subjects Specifications

Forty males participated in this study. The subjects were equally divided into two groups: an NSLBP group and a healthy control group. The number of individuals in each group was estimated using the literature (COP displacement) (Claeys et al., 2011), as well as a G-Power statistical software (Gpower, 2019). The inclusion criteria for the NSLBP patients included being free of vestibular disorders, radiculopathy, neurological, or respiratory disease, in addition to any surgical procedures involving the spine, neck, chest, or lumbar. Demographic data was recorded including age, height, weight, and BMI index (Table 1). Prior to starting the experimental testing, each individual completed two questionnaires designed to assess LBP by ODI (Oswestry Disability Index) (Fairbank and Pynsent, 2000), and to rate back pain on a numerical scale by NPRS (quantization of pain), respectively (Joos et al., 1991). Individuals were then assigned to the “low back pain” group if they reported ODI > 6 or NPRS > 0. However, all men in the healthy cohort reported zero for both NPRS and ODI questionnaires in this study. If any participant reported any pain at the time of the test, it was postponed to a later date.

**TABLE 1:** Demographic data of healthy and low back patients participants.

Variables	Healthy	NSLBP	*p*-value
N (Gender)	20 (Male)	20 (Male)	
Age	25.5 (±0.7)	24.5 (±0.9)	NS
Height (cm)	174 (±6.5)	172 (±7.5)	NS
Weight (kg)	64 (±8.6)	62 (±7.5)	NS
BMI (kg/m^2^)	20.3 (±2.3)	21.7 (±2.4)	NS

### Muscle Proprioception

**FIGURE 1:**
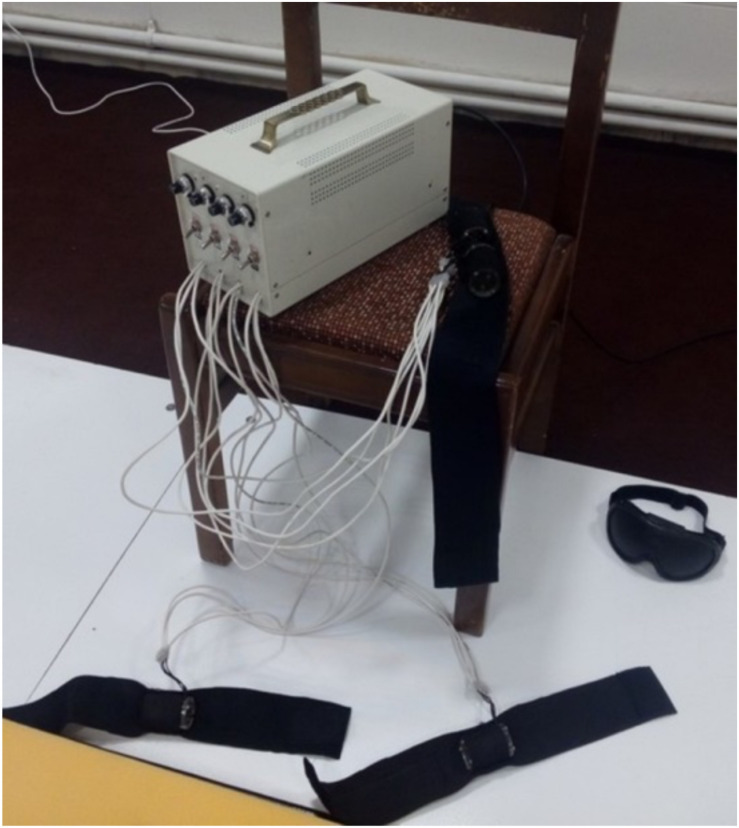
In-house vibrator apparatus for producing of muscle vibration.

There are several ways to alter proprioception input, the most common of which is to externally vibrate the muscles (Goodwin et al., 1972; Roll and Vedel, 1982). In order to alter proprioception of the soleus and lumbar muscles, we developed an in-house vibrator apparatus equipped with four brushless DC motors to produce muscle vibration (Figure 1). The device was placed at the longissimus and multifidus muscles spanning the lumbar vertebrae L3 to L5, as well as in the triceps surae located at the calf of the lower legs. Previous research suggests that optimal proprioception alteration occurs at a frequency of 70 Hz (Goodwin et al., 1972; Roll and Vedel, 1982; Cordo and Gurfinkel, 2004), while another reports a frequency of 60 Hz and an amplitude of 0.5 mm as ideal for altering one’s sense of proprioception (Claeys et al., 2011). The vibration frequency of our device was set to 70 Hz, with amplitude of about 0.5 mm to bias the proprioceptive data. When the vibrators were applied to the soleus muscles, an illusion of ankle dorsiflexion was externally induced. In response, the central nervous system (CNS) used the biased proprioceptive data to incline the body rearward to maintain balance. Conversely, when the vibrations were applied to the lumbar area, an illusion of extension was externally induced, causing the CNS to execute a forward incline.

### Procedure

**FIGURE 2:**
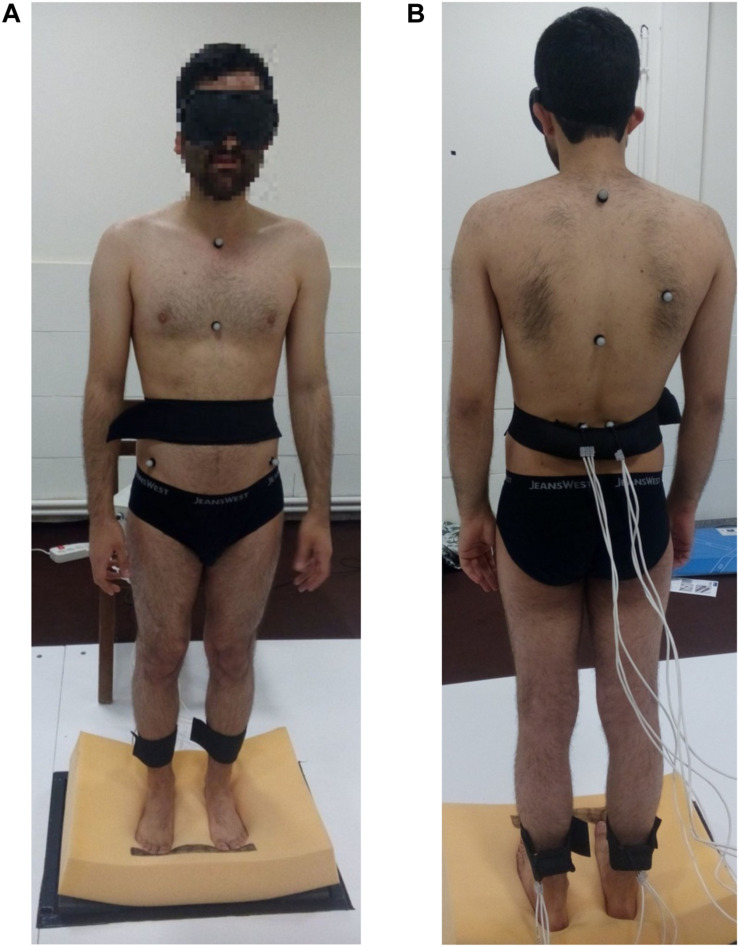
Exprimental set-up.

A force plate (Bertec, United States) was used to record the body’s center-of-pressure (COP) fluctuations and to obtain the trunk angles through inverse dynamics. A Vicon optical motion capture system with markers synced to the force plate was used in conjunction. The markers were positioned at the C7, T12, lower sternum (xiphoid process), clavicle (*Incisura jugularis*), right scapula, right and left sides of the PSIS (posterior superior iliac spine) and ASIS (anterior superior iliac spine) based on literature. The coordinate system was defined such that the axis perpendicular to the individual’s coronal plane was defined as the *X*-axis [anterior-posterior (AP)], the axis perpendicular to the sagittal plane was set as the *Y*-axis [medial-lateral (ML)], and the *Z*-axis [proximal distal (PD)] was perpendicular to the transverse plane. The selected sampling frequency on both devices was 100 Hz. The motor straps were attached to the end of triceps surae muscle on each foot, and on the multifidus muscles bilaterally. Each participant, with occluded vision (using am eye mask), performed 8 separate trials as follows: (1) standing on a motionless rigid surface (without any vibrator-induced movement); (2) standing on a rigid surface with the activation of the triceps vibrators; (3) standing on a rigid surface with the activation of the multifidus vibrators; (4) standing on a rigid surface with the activation of both the triceps and multifidus vibrators; (5) standing on a motionless foam surface; (6) standing on a foam surface with the activation of the triceps vibrators; (7) standing on a foam surface with the activation of the multifidus vibrators; and (8) standing on a foam surface with the activation of both the triceps and multifidus vibrators. Conditions were applied in random. For each trial, COP data was recorded in both the anterior posterior (AP) and medial lateral (ML) positions; trunk angles were also recorded in the three anatomical planes. Each trial lasted 30 s: (1) 10 s with the individual standing on the force place in the absence of any vibration (the balance phase); and (2) 20 s when the motors were turned on at a frequency of 70 Hz (the vibration phase). The experimental set-up in this study is shown in Figure 2.

### Filtering and Time Series Separation

**FIGURE 3:**
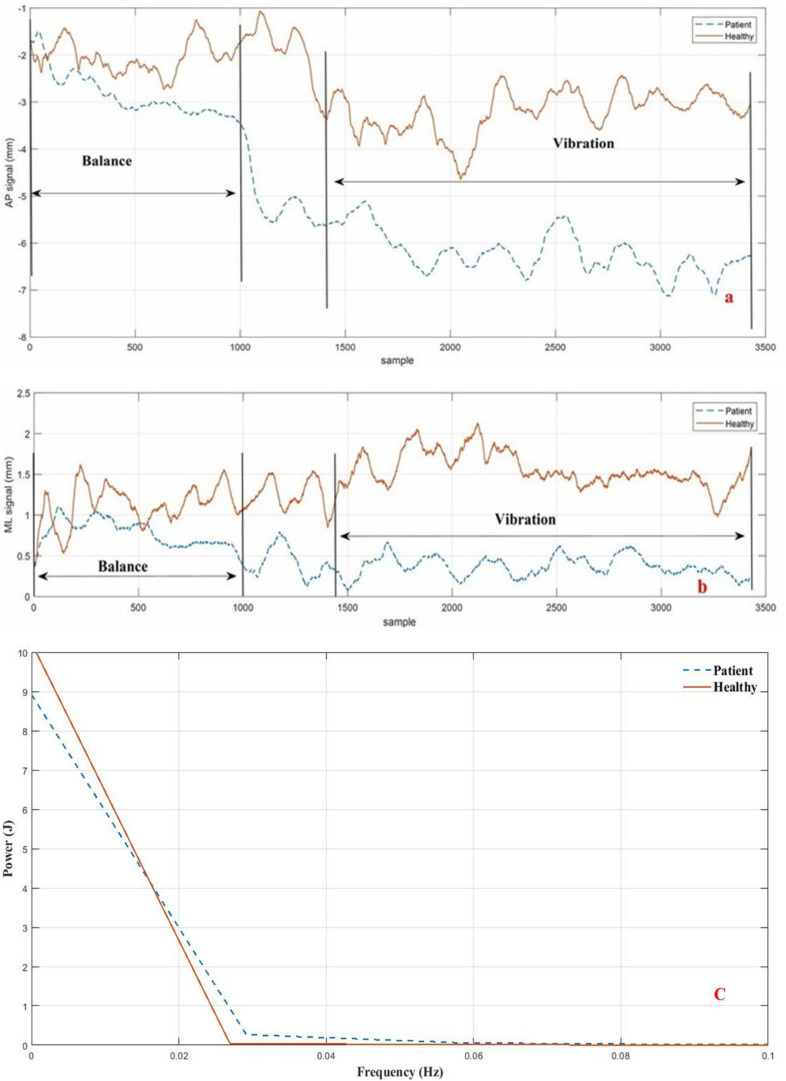
Divided signal and signal power of COP for a healthy subject and a LBP subject during Trial #2 (Ankle vibration on rigid surface). **(a)** AP direction; **(b)** ML direction; **(C)** signal power.

In order to filter COP and trunk angle data, the exact cutoff frequency was determined acoustically via spectral analysis. The amount of signal energy was determined in terms of the frequency. 99% of signal strength for all COP and trunk sagittal angles was at a frequency of less than 5 Hz; thus, the cutoff frequency of 5 Hz was used for data filtering (Figure 3C). The data was then filtered by selecting a second-order Butterworth non-linear filter, according to literature (Ghomashchi et al., 2011).

### Linear Analysis of COP Time Series

In order to analyze center-of-pressure data, the standard deviation of displacement, standard deviation of velocity, the mean total velocity, and the phase plane portrait for both anterior-posterior (AP) and medial-lateral (ML) directions were obtained according to Eq. 7–Eq. 14 Supplementary Appendix Table A1 (Salavati et al., 2009), in which $\bar{x}$ is the average of balance time series, *x*_*i*_ corresponds to each point of vibration time series, and *N* indicates the length of the time series. However, while the COP sway toward balance condition can be explained by linear analysis, it is usually not sufficiently powerful for a detailed kinematic interpretation of the physiological signal results. Thus, other non-linear tools were required, which are explained in the following sections.

### Non-linear Analysis of COP Time Series and Trunk Angle

#### Phase Space Reconstruction

The phase space for a dynamic system refers to a space in which all possible states are shown. Each possible state for the system represents a point in this space. Although there are several methods for analyzing the non-linear time series of a phase space for a dynamic system, the Time delay method is most commonly used. The most challenging step of this method is to identify (τ) Time Delay and (m) Embedding Dimension. For a time series of scalar variables according to Eq. 1:

(1) $$x\left(t_{i}\right),i=1,..,N$$

We can construct a vector in the phase space according to Eq. 2 at any time:

(2) $$X\,\left(t_{i}\right)=\left[x\,\left(t_{i}\right),x\,\left(t_{i}+\tau \right),x\,\left(t_{i}+2\,\tau \right),\ldots ,x\,\left(t_{i}+\left(m-1\right)\,\tau \right)\right]$$

Average mutual information (AMI) and false nearest neighbors (FNN) represent two standard methods for determining the time-delay parameter and the embedding dimension parameter, respectively (Horak et al., 2003). MATLAB software was used to reconstruct the phase space. For each individual, the phase space was reconstructed separately for each of the three signals: APCOP, MLCOP, and trunk angle. In most cases, the space embedding dimension for both the COP and trunk angle was 3. The time delay was assumed to be the first minimal relative for each person. Subsequently, the obtained phase space was verified using Chaos Data Analyzer software (Sprott, 1998), which confirmed the validity of the embedding dimension value. Time delay and embedding dimension values for COP and trunk data were assessed for each person individually and are summarized in Table 2.

**TABLE 2:** Embedding dimension and time delay values used as input parameters for phase space reconstruction of COP and trunk angle.

	COP		Trunk angle
	
	AP	ML	
Embedding Dimension	3 or 4	3 or 4	4 or 5
Time delay (sec)	0.35–0.6	0.35–0.6	0.1–0.2

*COP represents Center of Pressure. In addition, AP and Ml represent Anterior-Posterior and Mediolateral directions respectively. Also Trunk angle represents sagittal angle of trunk.*

#### RQA Method

Another prominent method for non-linear time series analysis is recurrence quantification analysis (RQA). Using this approach, the dynamic properties of a system’s path in a phase space can be represented in a two-dimensional space. Riley et al. (1999) expressed numerical criteria based on diagonal lines in n recurrence plot (RP), which can be used to analyze the amount of recurrence or complexity of the dynamics of an observed time series. In this study, RQA quantitative measurements were calculated using the RQA software (Webber, 2009), developed by Webber et al. (Webber and Zbilut, 2005). The Euclidean norm was used for calculating these criteria and the neighborhood radius was identified (Riley et al., 1999), which was considered 2.5% of the mean distance.

#### Short and Long Terms of Lyapunov

Next, the phase space for both the COP and trunk angle time series were reconstructed. *X*_$\bar{j}$_ can be determined by exploring through all points such that its distance from the reference *X*_$\bar{j}$_ is minimized, according to Eq. 3:

(3) $$d_{i}\,\left(0\right)=\min_{X_{\bar{j}}}∥X_{j}-X_{\bar{j}}∥$$

Where ∥…∥ is a Euclidean norm.

A Lyapunov function was used for both the COP (both directions) and trunk angle using Eq. 4:

(4) $$y\,\left(i\right)=\frac{1}{\Delta \,t}\,\left\langle \ln\left(d_{j}\,\left(i\right)\right)\right\rangle =\left[\lambda \right]\,i+c$$

Where ⟨…⟩ expresses the mean of the neighboring data points for all values of j. This function was divided by the sampling time intervals (Rosenstein et al., 1993). The short-term time (λ_*S*_) scale was obtained by the initial slope of the curve for the first few sampling intervals. Similarly, the long-term Lyapunov (λ_*L*_) exponent was obtained by the slope of the function after the rising interval. Positive values for the two exponents represent the divergence of the two neighboring paths of phase space (unstable), while negative values represent the convergence of the two neighboring paths– their combination expresses the relative stability of the system. Large and positive exponents are indicators of the system’s dynamic instability; conversely, the larger and negative the exponents, the greater the stability of the system. For this investigation, the slope of the Lyapunov function in the range of 1 to 30 samples determined the short-term Lyapunov, while the slope of the Lyapunov function in the range of 250–500 samples determined the long-term Lyapunov exponent for both the COP and trunk-angle time series.

### Statistical Analysis

The linear and non-linear analysis results of the COP and trunk data were compared using SPSS (SPSSsoftware, 2019), where analysis of variance (ANOVA) and multiple analysis of variance (MANOVA) were employed to check for significant differences. In this study, the independent variables consisted of the group category (healthy or NSLBP), the vibration covered muscular area (triceps, multifidus, none and both), and the foot placement condition (rigid or foam) 2×4×2. The results were considered significant at a level of *P* < 0.05. Subsequently, all dependent variables were subjected to multi-factor ANOVA, followed by a *post hoc* Tukey’s test using Bonferroni adjustment/correction of the independent variables (Field, 2013).

## Results

The results of ODI and NPRS questionnaires demonstrate significant differences between the healthy participants and the LBP group, as shown in Table 3.

**TABLE 3:** Oswestry disability inventory questionnaire and pain scale results from participants.

Questioners	Healthy (SD)	Patient (SD)	Significant difference
ODI-2 (0-100)	0	12.3(3.6)	Yes
NPRS (0-10)	0	2.5(1.2)	Yes

The recorded data associated with the force-plate testing was divided into two 10-s segments (balance part) and one 20-s segment (vibration part). Figures 3A,B show the results for the second segment in both directions (AP and ML), while the cutoff frequency (5 Hz) for the sample data is shown in Figure 3C with the person standing on the stationary rigid surface with active triceps vibrators.

**FIGURE 4:**
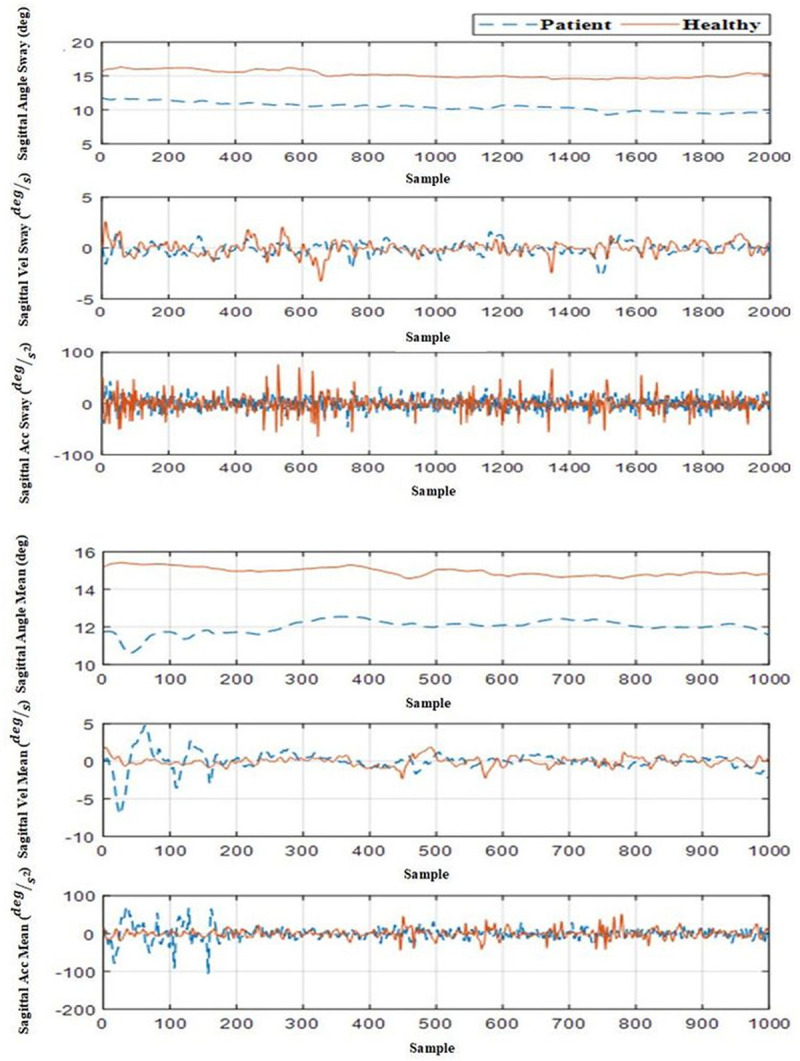
Angle, angular velocity, and angular acceleration of trunk in sagittal view for a healthy participant and a LBP participant in Trial #2 (ankle vibration on rigid surface).

The trunk kinematics (angular velocity and the angular acceleration) were obtained using sequential numerical derivatives of the trunk angular position as shown in Eq. 5–Eq. 6. Since the noise effects increase may impact RQA analysis, the derivate was filtered once again. On the other hand, subsequent RQA analyses of angular velocity and angular acceleration data showed unexpected results [positive trend (+1.2)], which we attribute to the noise effect. Therefore, while no analysis was conducted on the angular velocity and acceleration of the trunk, the effect of noise on angular velocity remains uncertain and cannot be factored out from the data analysis. The angular position, velocity and acceleration for the trial #2 are depicted in Figure 4 for both healthy and the LBP participants.

(5) $$\dot{\theta }\,\left(A\,n\,g\,u\,l\,a\,r\,V\,e\,l\,o\,c\,i\,t\,y\right)=\frac{d\,\theta }{d\,t}$$

(6) $$\ddot{\theta }\,\left(A\,n\,g\,u\,l\,a\,r\,A\,c\,c\,e\,l\,e\,r\,a\,t\,i\,o\,n\right)=\frac{d\,\dot{\theta }}{d\,t}$$

All linear indicators have been listed in Supplementary Appendix Tables A2, A3. Note that the values for the linear parameter data were higher in the LBP individuals as compared with the healthy control in both the AP and ML directions for the rigid and foam conditions with vibration. This finding indicates that to maintain balance, the LBP group altered their COP more than their healthy counterparts, which made them more reliant on the ankle proprioception strategy, thereby leading to increased COP variation. These changes were evident when the ankle vibrators were activated on the foam surface **(σ_x_ = Healthy 18.82 < Patient 28.91 and σ_υ_x__** = **Healthy 22.11 < Patient 29.21)**. Table 4 shows the results of the statistical analyses with linear parameters.

**TABLE 4:** Results of three way analysis of variance (ANOVA) tests for the effects of surface, vibration and group on the linear parameters of COP.

Independent Variable	σ_x_		σ_y_		σ_vx_		σ_vy_		σ_*rx*_		σ_ry_		V_Total_		σ_*r*_	
								
	*F*	*P*	*F*	*P*	*F*	*P*	*F*	*P*	*F*	*P*	*F*	*P*	*F*	*P*	*F*	*P*
**Main Effect**																
Surface	**11.06**	***P* < 0.05**	**81.75**	***P* < 0.05**	**199.67**	***P* < 0.05**	**162.43**	***P* < 0.05**	**246.19**	***P* < 0.05**	**521.18**	***P* < 0.05**	**277.97**	***P* < 0.05**	**163.57**	*P* < 0.05
Vibration	**53.43**	***P* < 0.05**	**14.32**	***P* < 0.05**	**6.35**	***P* < 0.05**	**18.9**	***P* < 0.05**	**57**	***P* < 0.05**	**67.76**	***P* < 0.05**	**24.38**	***P* < 0.05**	**33.67**	*P* < 0.05
Group	**69.02**	***P* < 0.05**	**259.8**	***P* < 0.05**	**36.56**	***P* < 0.05**	**84.57**	***P* < 0.05**	**157.6**	***P* < 0.05**	**583.19**	***P* < 0.05**	**72.53**	***P* < 0.05**	**118.54**	*P* < 0.05
**Interaction**																
Surface × Vibration	**2.48**	*P* = 0.06	**4.1**	***P* < 0.05**	**0.73**	*P* = 0.55	**2.49**	*P* = 0.06	**1.82**	*P* = 0.14	**9.4**	***P* < 0.05**	**5.72**	***P* < 0.05**	**1.28**	*P* = 0.28
Surface × Group	**3.38**	*P* = 0.06	**47.1**	***P* < 0.05**	**9.74**	***P* < 0.05**	**16.32**	***P* < 0.05**	**1.657**	*P* = 0.19	**108.51**	***P* < 0.05**	**1.035**	*P* = 0.31	**4.39**	***P* < 0.05**
Vibration × Group	**12**	***P* < 0.05**	**8.8**	***P* < 0.05**	**1.21**	*P* = 0.3	**5.79**	***P* < 0.05**	**11.61**	***P* < 0.05**	**26.74**	***P* < 0.05**	**3.27**	***P* < 0.05**	**7.72**	***P* < 0.05**
Surface × Vibration × Group	**1.66**	*P* = 0.17	**3.5**	***P* < 0.05**	**0.05**	*P* = 0.98	**2.21**	*P* = 0.08	**0.98**	*P* = 0.39	**7.77**	***P* < 0.05**	**0.58**	*P* = 0.62	**0.25**	*P* = 0.85

*All bold values are significant at *p* < 0.05.*

The RQA parameters for both the AP and ML directions of COP are shown in Supplementary Appendix Tables A4, A5). Note that the value of Recurrence in the LBP cohort, as compared to the healthy group, indicates the presence of repetitive points and more repetitive sway in motor behavior, especially on foam. This was evident in the trials performed with the active vibrators **(0.45 > 0.11)**. Furthermore, the value of Determinism was greater in the LBP group as compared to the healthy individuals. This was more remarkable when the triceps vibrators were activated, especially on foam **(99.52 > 96.44)**, suggesting the reliance on more repetitive patterns among the LBP group.

**FIGURE 5:**
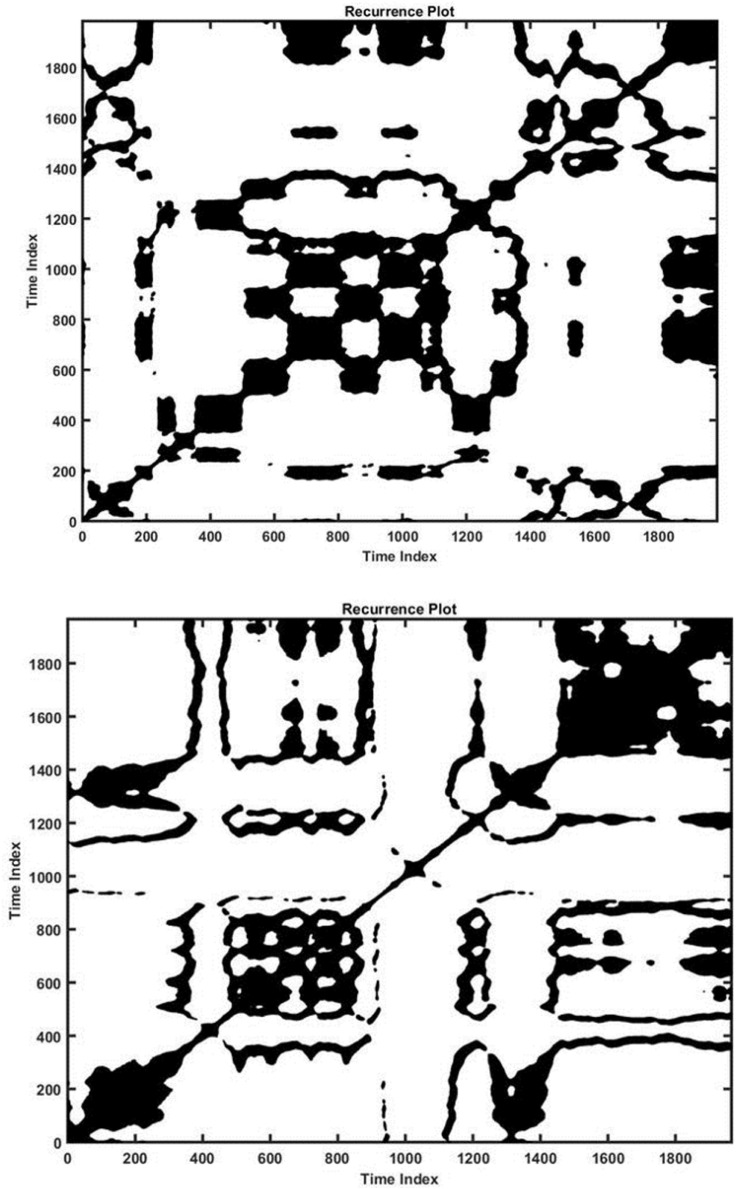
Recurrence plot for a healthy (down) and a LBP (up) individual in Trial #2 (ankle vibration on foam surface).

The Entropy value, which expresses the complexity of determinism, was also calculated. Entropy was higher for the LBP group as compared to the healthy group across most of the trials **(4.69 > 3.9)**. The trend is also shown in Supplementary Appendix Tables A4, A5, which helps explain the non-stationary behavior of the system. Specifically, the amplitude of this parameter was higher in the LBP group than the healthy individuals, especially on foam with muscles vibration **(−0.89 > −0.2)**. The full statistical analysis of the RQA parameters is shown in Supplementary Appendix Table A6, where most of these parameters indicate significant differences between the LBP and Healthy cohort **(*P* < 0.05)**. Results for the RQA parameters of the trunk data are provided in Supplementary Appendix Tables A7, A8. RQA measures based on diagonal lines including Recurrence, determinism, entropy, and trend for each group of the COP time series were calculated from the recurrence plots, as shown in Figure 5 for both cohorts (Trial #6). The concept of RQA parameters and their relationship with the diagonal lines can be found in van den Hoorn et al. (2018). The results of the statistical analyses are provided in Supplementary Appendix Table A9.

Short-term and long-term Lyapunov exponents are shown in Supplementary Appendix Tables A10, A11 for the COP and trunk angle data. For all the trials, the phase space path stability of the healthy cohort was higher than that of the LBP cohort (less Lyapunov exponents value). These results are consistent with the results of the velocity deviation parameters for both the AP and ML directions as shown in Supplementary Appendix Tables A2, A3. Moreover, it seems that there is an association between instability and the increase of velocity deviation in both cohorts. It can be seen from the short and long-term Lyapunov exponents that the LBP individuals experienced greater problems with stability in comparison with the healthy group under the same testing conditions. Moreover, when the same tests were conducted on the softer surface (foam), those instability differences became more pronounced **(λ_s_** = **Healthy 2.5 < Patient 3.2 and σ_υx_** = **Healthy 22.11 < Patient 29.21)**. Statistical analysis of Lyapunov Exponents are provided in Supplementary Appendix Table A12, where short-term Lyapunov shows more significant differences between LBP and Healthy cohorts as compared to long-term Lyapunov (*P* < 0.05).

## Discussion

This work presents a quantitative methodology that leverages both linear and non-linear dynamic tools to delineate and discriminate proprioception control in non-specific low back pain patients as compared to healthy individuals.

The linear analysis employed here revealed that the standard deviation of amplitude and velocity of the COP were higher among the LBP group as compared to the healthy controls in both AP and ML directions, suggesting that the LBP patients experienced a greater challenge in using the hip control strategy to maintain stability instead of the ankle strategy. This was most apparent in the trials during which the vibrators were active (Trials 8, 7, 6, 4, 3, and 2 as stated in the above procedure) and while standing on the foam surface. These findings are consistent with previous research (Brumagne et al., 2008). However, it is not clear whether this change of strategy in the LBP cohort is due to a disorder in lumbar proprioception receptors making them unable to send the proprioception data to the brain correctly, or whether the control scheme of the brain is actually altered by the LBP, causing the brain to use less of these data (della Volpe et al., 2006).

The non-linear dynamic analysis, including the analysis of the COP data in terms of recurrence, determinism and entropy in the both directions showed that the LBP individuals have more repetitive patterns and sway as compared to the healthy group. This renders them less able to adapt to the environmental conditions and use repetitive sway behavior to maintain stability, particularly while on the foam surface which requires more flexibility and adaptive control behavior. Trend, or the measure of the non-stationary behavior of a system, was shown to be higher among the LBP group, reflecting failure to achieve a balance point. In conjunction with an increase in the standard deviation of the COP, this may be interpreted as functional brain changes that occur during proprioceptive processing in LBP patients contributing to their postural control impairments. Hence, when the brain is challenged to identify a specific equilibrium point, it may compensate for this lack of adjustment by increasing the variability of the COP to obtain equilibrium (Ghomashchi et al., 2011).

Functional stability analyses (Supplementary Appendix Tables A10, A11) based on short-term and long-term Lyapunov stability components demonstrated a higher short-term exponent in the LBP cohort as compared to the healthy group for the COP and trunk data. This indicates reduced stability in LBP individuals, suggesting that these patients are less likely to use their full body potential to maintain stability and instead rely more on their ankle joints. This adaptive control strategy is probably due to the less flexible lumbar area as compared to healthy people.

Statistical analyses indicated that for most of the parameters used in this study (linear parameters, RQA and Lyapunov components), there were significant differences between the LBP cohort and the healthy group. This suggests that the methodology introduced here (Linear and Non-linear indicators) along with the various quantitative parameters could be incorporated in the diagnosis and treatment/rehabilitation of individuals with proprioception disorders, including LBP patients. Specifically, physiotherapists should consider the increased use of therapeutic exercises that encourage the use of hip strategy for maintaining stability and to prevent LBP recurrence. The less complexity in NSLBP behaviors (Supplementary Appendix Tables A4, A5) can be explained by their higher muscle co-activation (Guthart and Salisbury, 2000) and higher reliance on the ankle strategy (Brumagne et al., 2008) that may reduce the stabilizing control in the ML direction.

A number of limitations must be acknowledged. First, in the absence of a device such as gyroscope and accelerometer to obtain direct angular velocity and angular acceleration of the trunk, we relied on a derivative method for calculating these two parameters, which could have led to unreliable results in analyzing and interpreting the data. Second, we did not employ a direct questionnaire or experimental trial that could have unequivocally identified those with proprioception disorders, the patients self-identified which may have affected the results. While motor control adaptation in LBP has been extensively studied from a motor output perspective, much less attention has been paid to changes in sensory input, specifically proprioception. Future studies are needed to use the quantitative tools proposed here to further investigate the adaptive strategies and their impact on the chronification of LBP.

## Conclusion

This study developed a methodology that leverages linear and non-linear dynamic tools to quantitatively study proprioception impairment in a cohort of LBP patients. The linear analyses results indicated an increase of the standard deviation of amplitude and velocity among the LBP participants, reflecting that these patients were mechanically challenged while using a hip control strategy to maintain stability, and hence opted for an ankle control strategy instead. Non-linear analyses of recurrence, determinism, and entropy from the COP in both directions, coupled with the trunk kinematic data, demonstrated that the LBP participants used more repetitive sway kinematics, as compared to their healthy counterparts, reflecting diminished adaptive capability to environmental conditions. Higher trend values in the LBP group indicated that they engage in more non-stationary sway behaviors. The short-term Lyapunov component was greater in the LBP group suggesting greater physical instability. From a short-term perspective, our work suggests that LBP patients tend not to use their full body potential to maintain stability and instead rely on the ankle control strategy, possibly due to a compromised or less flexible lumbar area and/or fear of further injury. Future studies are needed to investigate the long-term impact of impaired proprioceptive signaling and its role in postural control.

## Data Availability Statement

The raw data supporting the conclusions of this article will be made available by the authors, without undue reservation.

## Ethics Statement

The studies involving human participants were reviewed and approved by the IRB of Shahid Beheshti University of Medical Sciences, Tehran, IR, No: IR.SBMU.RETECH.REC.1396.1392). The patients/participants provided their written informed consent to participate in this study.

## Author Contributions

MP, MH, and KK: conceptualization, design, and coordination the study. MS, MA, and SBr: methodology and data analysis. MD: writing-original draft preparation. MH, KK, and SS: writing-review and editing. All authors have read and agreed to the published version of the manuscript.

## Conflict of Interest

The authors declare that the research was conducted in the absence of any commercial or financial relationships that could be construed as a potential conflict of interest.
