# Supplementary material for: Linear and Non-linear Dynamic Methods Toward Investigating Proprioception Impairment in Non-specific Low Back Pain Patients
Source: Front Bioeng Biotechnol. 2020 Nov 30;8:584952. doi: 10.3389/fbioe.2020.584952 (PMC7734295; doi:10.3389/fbioe.2020.584952)
Supplement: Supplementary file 1 [file Presentation_1.pdf]

Appendix

Table A1: linear parameters of COP analysis (Salavati et al., 2009)

Parameter	Formula
SD of Amplitude (mm)	
AP Eq. 1	$\sigma_x = \sqrt{\frac{\sum (x_i - \bar{x})^2}{N-1}}$
ML (Eq. 2)	$\sigma_y = \sqrt{\frac{\sum (y_i - \bar{y})^2}{N-1}}$
SD of Amplitude (mm/s)	
AP (Eq. 3)	$\sigma_{v_x} = \sqrt{\frac{\sum (v_{x_i} - \bar{v})^2}{N-1}}$ $v_{x_i} = \frac{x_{(i+1)} - x_i}{t_{(i+1)} - t_i}$
ML (Eq. 4)	$\sigma_{v_y} = \sqrt{\frac{\sum (v_{y_i} - \bar{v})^2}{N-1}}$ $v_{y_i} = \frac{y_{(i+1)} - y_i}{t_{(i+1)} - t_i}$
mean total velocity (mm/s)	
(Eq. 5)	$\bar{V} = \frac{1}{T}\sum_{i=1}^{T}\sqrt{(x_{i+1} - x_i)^2 + (y_{i+1} - y_i)^2}$
Phase plane portrait (Arbitrary unit)	
AP (Eq. 6)	$\sigma_{rx} = \sqrt{\sigma_x^2 + \sigma_{v_x}^2}$
ML (Eq. 7)	$\sigma_{ry} = \sqrt{\sigma_y^2 + \sigma_{v_y}^2}$
AP-ML Eq. 8	$\sigma_r = \sqrt{\sigma_{r_x}^2 + \sigma_{r_y}^2}$

Unit of measures are as follow: σ_x and σ_y are in mm, σ_{v_x} σ_{v_y} ,V_{Total} are in $mm/_s$, σ_{r_x} ,σ_{r_y} and σ_r are in arbitary unit

Table A2 : Descriptive data for linear parameters of COP on rigid suface

Variables	Without Vibration		Ankle Vibration		Back Vibration		Both Vibrations	
	**Healthy**	**Patient**	**Healthy**	**Patient**	**Healthy**	**Patient**	**Healthy**	**Patient**
$\sigma_x(SD)$	**9.35(3.32)**	**8.01(2.29)**	**12.82(3.95)**	**23.31(9.88)**	**7.03(2.43)**	**14.92(6.51)**	**10.3(4)**	**23.56(8.25)**
$\sigma_y(SD)$	**3.06(1.26)**	**4.56(1.87)**	**3.67(1.31)**	**7.2(3.24)**	**2.76(1.09)**	**6.67(2.85)**	**2.65(1.22)**	**7.62(3.51)**
$\sigma_{v_x}(SD)$	**11.9(4.09)**	**12.32(4.16)**	**13.5(4.66)**	**15.92(6.79)**	**8.52(3.36)**	**12.4(4.97)**	**13.92(3.96)**	**16.21(5.51)**
$\sigma_{v_y}(SD)$	**5.71(1.79)**	**6.58(2.25)**	**6.56(1.22)**	**8.71(2.74)**	**3.6(1.21)**	**5.88(2.03)**	**6.95(2.61)**	**9.2(3.2)**
$\sigma_{r_x}(SD)$	**15.15(3.48)**	**14.71(3.62)**	**18.61(3.94)**	**28.21(5.79)**	**11.04(2.61)**	**19.39(3.14)**	**17.32(3.14)**	**28.64(7.13)**
$\sigma_{r_y}(SD)$	**6.48(1)**	**8.02(1.81)**	**7.52(1.24)**	**11.31(2.14)**	**4.54(1.02)**	**8.89(1.99)**	**7.44(1.32)**	**11.94(2.24)**
$V_{Total}(SD)$	**10.22(2.98)**	**11.42(3.09)**	**11.32(3.6)**	**16.64(4.3)**	**7.55(1.89)**	**11.04(2.18)**	**12.67(3.16)**	**18.24(5.37)**
$\sigma_r(SD)$	**15.92(4.85)**	**7.56(2.29)**	**12.62(3.95)**	**23.1(9.88)**	**6.73(2.43)**	**14.43(6.51)**	**9.92(4)**	**23.06(8.25)**

Table A3 : Descriptive data for linear parameters of COP on foam suface

Variables	Without Vibration		Ankle Vibration		Back Vibration		Both Vibrations	
	Healthy	Patient	Healthy	Patient	Healthy	Patient	Healthy	Patient
$\sigma_x\,(SD)$	9.52(4.61)	8.45(2.76)	18.82(8.5)	28.91(11.84)	10.61(3.19)	15.86 (5.64)	15.98(7.44)	21.03(10.53)
$\sigma_y\,(SD)$	3.5(0.93)	8.1(2.69)	4.01(1.71)	16.82(7.75)	3.1(1.47)	10.75(4.37)	4.8(2.04)	14.3(6.58)
$\sigma_{v_x}\,(SD)$	19.58(7.08)	23.86(9.28)	22.11(7.66)	29.21 (10.78)	18.62(6.63)	27.59(11.36)	21.45(6.07)	29.38(7.37)
$\sigma_{v_y}\,(SD)$	8.81(3.04)	9.5(1.97)	10.14(3.28)	15.5(4.41)	7.2(1.15)	13.59(5.16)	9.42(3.46)	16.38(6.95)
$\sigma_{r_x}\,(SD)$	21.76(4.1)	25.31(5.7)	29.01(7.32)	41.13(9.04)	21.42(4.85)	31.82(6)	26.79(6.44)	36.14(9.26)
$\sigma_{r_y}\,(SD)$	9.47(1.34)	12.5(2.18)	10.91(2.03)	22.9(4.72)	7.84(1.59)	17.33(3.02)	10.57(2.11)	21.74(3.68)
$V_{Total}\,(SD)$	15.62(2.93)	18.33(3.09)	21.32(6.21)	25.35(6.42)	18.32(5.36)	23.9(5.28)	19.03(4.54)	26.52(7.02)
$\sigma_r\,(SD)$	23.46(5.24)	27.83(8.12)	30.74(8.11)	46.73(13.66)	22.36(5.92)	35.91(11.97)	28.24(9.14)	41.62(14.33)

Table A4 : Descriptive data for RQA parameters of COP on rigid surface

Variables	Without Vibration		Ankle Vibration		Back Vibration		Both Vibrations	
	Healthy	Patient	Healthy	Patient	Healthy	Patient	Healthy	Patient
$\mathrm{Reccurence}_{\_AP}\,(SD)$	0.32(0.17)	0.24(0.1)	0.14(0.08)	0.28(0.15)	0.17(0.09)	0.23(0.13)	0.24(0.12)	0.18(0.1)
$\mathrm{Reccurence}_{\_ML}\,(SD)$	0.22(0.14)	0.13(0.08)	0.12(0.06)	0.23(0.16)	0.12(0.06)	0.27(0.17)	0.17(0.07)	0.21(0.12)
$Determinism_{\_AP}\,(SD)$	99.84(1.25)	99.81(1.56)	98.81(2.16)	99.06(1.16)	99.53(2.08)	99.6(1.53)	99.81(1.46)	99.79(1.25)
$Determinism_{\_ML}\,(SD)$	99.65(1.37)	99.42(1.56)	98.28(1.41)	99.33(1.77)	98.38(1.2)	99.71(1.3)	99.76(1.52)	99.5(1.42)
$Entropy_{\_AP}\,(SD)$	4.85(0.56)	4.6(0.44)	4.17(0.53)	3.85(0.4)	4.47(0.41)	4.31(0.51)	4.13(0.44)	4.46(0.5)
$Entropy_{\_ML}\,(SD)$	4.2(0.56)	4.28(0.44)	4 (0.38)	4.2(0.48)	3.9(0.36)	4.05(0.51)	4.13(0.55)	4.57(0.46)
$Trend_{\_AP}\,(SD)$	-0.62(0.23)	-0.47(0.17)	-0.28(0.1)	-0.54(0.18)	-0.33(0.13)	-0.46(0.14)	-0.48(0.18)	-0.35(0.13)
$Trend_{\_ML}\,(SD)$	-0.44(0.2)	-0.25(0.08)	-0.23(0.08)	-0.46(0.21)	-0.23(0.07)	-0.53(0.17)	-0.33(0.13)	-0.43(0.16)

Table A5: Descriptive data for RQA parameters of COP on foam surface

Variables	Without Vibration		Ankle Vibration		Back Vibration		Both Vibrations	
	Healthy	Patient	Healthy	Patient	Healthy	Patient	Healthy	Patient
$Reccurence_{\_AP}(SD)$	0.13(0.06)	0.1(0.05)	0.11(0.07)	0.45(0.28)	0.15(0.09)	0.19(0.09)	0.09(0.05)	0.18(0.13)
$Reccurence_{\_ML}(SD)$	0.16(0.09)	0.19(0.12)	0.14(0.08)	0.4(0.25)	0.21(0.11)	0.33(0.17)	0.15(0.05)	0.18(0.1)
$Determinism_{\_AP}(SD)$	99.5(1.36)	99.1(1.68)	96.44(1.48)	99.52(1.13)	97.67(1.11)	99.49(1.81)	98.26(1.67)	99.54(1.77)
$Determinism_{\_ML}(SD)$	99.41(1.89)	99.64(1.65)	98.53(1.8)	98.83(1.33)	99.74(1.8)	98.63(1.59)	98.63(1.59)	98.93(1.14)
$Entropy_{\_AP}(SD)$	4.2(0.5)	4.07(0.5)	3.9(0.42)	4.69(0.57)	3.9(0.47)	4.39(0.44)	4(0.43)	4.49(0.52)
$Entropy_{\_ML}(SD)$	4.61(0.59)	4.7(0.36)	4.11 (0.54)	4.03(0.38)	4.25(0.5)	4.63(0.48)	4.26(0.42)	3.95(0.34)
$Trend_{\_AP}(SD)$	-0.26(0.1)	-0.18(0.07)	-0.2(0.07)	-0.89(0.37)	-0.3(0.09)	-0.35(0.13)	-0.17(0.07)	-0.35(0.09)
$Trend_{\_ML}(SD)$	-0.31(0.12)	-0.38(0.18)	-0.26(0.1)	-0.76(0.32)	-0.41(0.15)	-0.66(0.25)	-0.3(0.12)	-0.34(0.08)

Table A6 : Results of Three way Analysis of Variance (ANOVA) tests for the effects of Surface, Vibration and Group on the RQA parameters of COP

Independent Variable	$Reccurence_{\_AP}$		$Reccurence_{\_ML}$		$Determinism_{\_AP}$		$Determinism_{\_ML}$		$Entropy_{\_AP}$		$Entropy_{\_ML}$		$Trend_{\_AP}$		$Trend_{\_ML}$	
	F	P	F	P	F	P	F	P	F	P	F	P	F	P	F	P
**Main Effect**																
surface	12.51	P<0.05	6.35	P<0.05	36.97	P<0.05	0.08	P=0.76	6.18	P<0.05	8.16	P<0.05	32.47	P<0.05	11.88	P<0.05
vibration	5.1	P<0.05	4.2	P<0.05	3.36	P<0.05	3.82	P<0.05	4.87	P<0.05	8.19	P<0.05	11.43	P<0.05	8.82	P<0.05
Group	19.96	P<0.05	31.55	P<0.05	9.28	P<0.05	6.04	P<0.05	6.77	P<0.05	4.96	P<0.05	42.89	P<0.05	73.83	P<0.05
**Interaction**																
surface× vibration	12.11	P<0.05	3.97	P<0.05	3.83	P<0.05	2.55	P=0.056	10.91	P<0.05	10.66	P<0.05	27.52	P<0.05	8.87	P<0.05
Surface× Group	11.53	P<0.05	4.09	P<0.05	0.9	P=0.34	1.94	P=0.16	19.67	P<0.05	3.4	P=0.06	24.84	P<0.05	7.84	P<0.05
vibration × Group	20.14	P<0.05	11.16	P<0.05	19.32	P<0.05	0.76	P=0.51	6.11	P<0.05	0.85	P=0.46	48.19	P<0.05	26	P<0.05
surface× vibration× Group	3.09	P<0.05	2.43	P=0.06	14.28	P<0.05	1.31	P=0.27	4.71	P<0.05	4.08	P<0.05	9.95	P<0.05	5.99	P<0.05

Table A7 : Descriptive data for RQA parameters of trunk angle on rigid surface

Variables	Without Vibration		Ankle Vibration		Back Vibration		Both Vibrations	
	Healthy	Patient	Healthy	Patient	Healthy	Patient	Healthy	Patient
$Recc_{\_Angle}(SD)$	0.18(0.1)	0.12(0.06)	0.41(0.24)	0.4(0.17)	0.14(0.08)	0.28(0.16)	0.15(0.1)	0.33(0.17)
$Det_{\_Angle}(SD)$	98.37(1.29)	99.33(1.57)	98.66(1.61)	99.66(1.66)	98.65(1.9)	98.41(1.44)	97.91(1.24)	99.45(1.44)
$Entropy_{\_Angle}(SD)$	3.92(0.37)	4.01(0.39)	3.89(0.37)	4.36(0.35)	3.84(0.56)	3.91(0.52)	3.49(0.28)	4.26(0.5)
$Trend_{\_Angle}(SD)$	-0.34(0.14)	-0.25(0.1)	-0.79(0.27)	-0.77(0.3)	-0.28(0.11)	-0.53(0.22)	-0.3(0.11)	-0.66(0.24)

Table A8 : Descriptive data for RQA parameters of trunk angle on foam surface

Variables	Without Vibration		Ankle Vibration		Back Vibration		Both Vibrations	
	Healthy	Patient	Healthy	Patient	Healthy	Patient	Healthy	Patient
$Recc_{\_Angle}(SD)$	0.18(0.07)	0.14(0.09)	0.18(0.07)	0.3(0.14)	0.1(0.06)	0.17(0.09)	0.16(0.1)	0.12(0.06)
$Det_{\_Angle}(SD)$	97.14(1.84)	98.51(1.82)	97.93(1.13)	98.95(1.29)	97.33(1.09)	97.89(1.95)	97.92(1.57)	98.89(1.68)
$Entropy_{\_Angle}(SD)$	3.58(0.41)	3.09(0.42)	3.68(0.51)	4.73(0.53)	3.8(0.36)	3.8(0.43)	3.79(0.39)	4.25(0.3)
$Trend_{\_Angle}(SD)$	-0.34(0.13)	-0.27(0.07)	-0.34(0.11)	-0.59(0.23)	-0.2(0.08)	-0.31(0.11)	-0.31(0.12)	-0.26(0.1)

Table A9 : Results of Three way Analysis of Variance (ANOVA) tests for the effects of Surface, Vibration and Group on the RQA parameters of Trunk angle

Independent Variable	$Recc_{\_Angle}$		$Det_{\_Angle}$		$Entropy_{\_Angle}$		$Trend_{\_Angle}$	
	F	P	F	P	F	P	F	P
**Main Effect**								
surface	34.64	P<0.05	17.73	P<0.05	8.91	P<0.05	71.7	P<0.05
vibration	29.77	P<0.05	3.15	P<0.05	1.16	P=0.32	57.89	P<0.05
Group	10.2	P<0.05	26.35	P<0.05	33.78	P<0.05	23.09	P<0.05
**Interaction**								
surface× vibration	6.65	P<0.05	0.9	P=0.43	6.17	P<0.05	12.22	P<0.05
Surface× Group	1.54	P=0.21	0.23	P=0.63	2.16	P=0.14	2.8	P=0.09
vibration × Group	5.7	P<0.05	2.07	P=0.1	6.43	P<0.05	9.43	P<0.05
surface× vibration× Group	7.01	P<0.05	0.7	P=0.55	2.27	P=0.08	13.78	P<0.05

Table A10 : Lyapunov Exponents for COP and trunk data on rigid surface

Variables	Without Vibration		Ankle Vibration		Back Vibration		Both Vibrations	
	Healthy	Patient	Healthy	Patient	Healthy	Patient	Healthy	Patient
$\lambda_{S\_AP}(SD)$	2.1(0.97)	2.4(1.02)	2.5(1.04)	3.2(1.35)	1.8(0.57)	2.76(1.17)	2.3(0.72)	2.7(1.15)
$\lambda_{L\_AP}(SD)$	0.05(0.03)	0.07(0.03)	0.18(0.09)	0.23(0.1)	0.06(0.02)	0.1(0.06)	0.13(0.07)	0.19(0.11)
$\lambda_{S\_ML}(SD)$	1.75(0.58)	1.89(0.7)	2.4(0.88)	2.9(1.08)	1.56(0.75)	2.71(1.25)	2.1(0.62)	2.74(0.77)
$\lambda_{L\_ML}(SD)$	0.05(0.02)	0.07(0.04)	0.13(0.06)	0.19(0.1)	0.07(0.03)	0.11(0.06)	0.1(0.06)	0.1(0.05)
$\lambda_{S\_Angle}(SD)$	1.78(0.74)	1.85(0.63)	2.5(0.47)	3.2(1.17)	1.2(0.57)	2.1(0.81)	2.2(0.77)	2.8(1.07)
$\lambda_{L\_Angle}(SD)$	0.08(0.04)	0.07(0.04)	0.35(0.19)	0.46(0.23)	0.15(0.06)	0.25(0.08)	0.29(0.15)	0.36(0.19)

Table A11 : Lyapunov Exponents for COP and trunk data on foam surface

Variables	Without Vibration		Ankle Vibration		Back Vibration		Both Vibrations	
	Healthy	Patient	Healthy	Patient	Healthy	Patient	Healthy	Patient
$\lambda_{S\_AP}(SD)$	3.21(1.11)	4.31(1.71)	3.85(1.24)	6.3(2.16)	3.36(1.53)	5.71(1.5)	3.78(1.43)	6.5(2.65)
$\lambda_{L\_AP}(SD)$	0.2(0.1)	0.25(0.15)	0.35(0.28)	0.45(0.29)	0.27(0.17)	0.32(0.17)	0.33(0.19)	0.41(0.16)
$\lambda_{S\_ML}(SD)$	2.81(1.16)	3.7(1.29)	3.4(1.32)	4.81(1.98)	2.1(0.85)	2.2(0.89)	3.5(1.07)	3.8(1.54)
$\lambda_{L\_ML}(SD)$	0.29(0.14)	0.3(0.17)	0.35(0.21)	0.42(0.22)	0.2(0.03)	0.34(0.25)	0.33(0.15)	0.38(0.25)
$\lambda_{S\_Angle}(SD)$	2.5(0.91)	2.7(0.97)	3.4(1.38)	4.2(1.44)	1.8(0.58)	3.2(0.95)	3.1(1.04)	3.9(1.57)
$\lambda_{L\_Angle}(SD)$	0.11(0.07)	0.13(0.08)	0.54(0.43)	0.76(0.43)	0.2(0.08)	0.25(0.14)	0.45(0.21)	0.69(0.46)

Table A12 : Results of Three way Analysis of Variance (ANOVA) tests for the effects of Surface, Vibration and Group on the Lyapunov Exponents of COP and Trunk angle

**Independent Va**	λ_{S_AP}		λ_{L_AP}		λ_{S_ML}		λ_{L_ML}		λ_{S_Angle}		λ_{L_Angle}	
	**F**	**P**	**F**	**P**	**F**	**P**	**F**	**P**	**F**	**P**	**F**	**P**
**Main Effect**												
surface	**181.52**	**P<0.05**	**130.73**	**P<0.05**	**69.01**	**P<0.05**	**193.66**	**P<0.05**	**64.53**	**P<0.05**	**29.98**	**P<0.05**
vibration	**7.33**	**P<0.05**	**17.92**	**P<0.05**	**19.17**	**P<0.05**	**7.85**	**P<0.05**	**29.47**	**P<0.05**	**60.84**	**P<0.05**
Group	**73.83**	**P<0.05**	**10.73**	**P<0.05**	**26.88**	**P<0.05**	**9.21**	**P<0.05**	**37.46**	**P<0.05**	**15.21**	**P<0.05**
**Interaction**												
Surface × vibration	**2.2**	P=0.08	**0.41**	P=0.74	**7.63**	**P<0.05**	**0.98**	P=0.39	**0.19**	P=0.9	**5.62**	**P<0.05**
Surface× Group	**23.88**	**P<0.05**	**0.66**	P=0.41	**0.07**	P=0.78	**1.4**	P=0.23	**1.08**	P=0.3	**1.66**	**P=0.19**
vibration × Group	**1.98**	P=0.11	**0.31**	P=0.81	**0.77**	P=0.5	**1.15**	P=0.32	**3.49**	P=0.14	**2.14**	**P=0.09**
surface× vibration × Group	**0.98**	P=0.4	**0.06**	P=0.98	**3.5**	P=0.08	**0.59**	P=0.62	**0.16**	P=0.92	**0.89**	**P=0.44**
